# Obesity related methylation changes in DNA of peripheral blood leukocytes

**DOI:** 10.1186/1741-7015-8-87

**Published:** 2010-12-21

**Authors:** Xiaoling Wang, Haidong Zhu, Harold Snieder, Shaoyong Su, David Munn, Gregory Harshfield, Bernard L Maria, Yanbin Dong, Frank Treiber, Bernard Gutin, Huidong Shi

**Affiliations:** 1 Georgia Prevention Institute, Department of Pediatrics, Medical College of Georgia, Augusta, GA, USA; 2 Department of Pediatrics, Medical College of Georgia, Augusta, GA, USA; 3 The Child Health Discovery Institute, Medical College of Georgia, Augusta, GA, USA; 4 The Cancer Research Center, Medical College of Georgia, Augusta, GA, USA; 5 Unit of Genetic Epidemiology and Bioinformatics, Department of Epidemiology, University Medical Center Groningen, University of Groningen, The Netherlands; 6 Department of Medicine, Division of Cardiology, Emory University School of Medicine, Atlanta, GA, USA

## Abstract

**Background:**

Despite evidence linking obesity to impaired immune function, little is known about the specific mechanisms. Because of emerging evidence that immune responses are epigenetically regulated, we hypothesized that DNA methylation changes are involved in obesity induced immune dysfunction and aimed to identify these changes.

**Method:**

We conducted a genome wide methylation analysis on seven obese cases and seven lean controls aged 14 to 18 years from extreme ends of the obesity distribution and performed further validation of six CpG sites from six genes in 46 obese cases and 46 lean controls aged 14 to 30 years.

**Results:**

In comparison with the lean controls, we observed one CpG site in the UBASH3A gene showing higher methylation levels and one CpG site in the TRIM3 gene showing lower methylation levels in the obese cases in both the genome wide step (*P *= 5 × 10^-6 ^and *P *= 2 × 10^-5 ^for the UBASH3A and the TRIM3 gene respectively) and the validation step (*P *= 0.008 and *P *= 0.001 for the UBASH3A and the TRIM3 gene respectively).

**Conclusions:**

Our results provide evidence that obesity is associated with methylation changes in blood leukocyte DNA. Further studies are warranted to determine the causal direction of this relationship as well as whether such methylation changes can lead to immune dysfunction.

See commentary: http://www.biomedcentral.com/1741-7015/8/88/abstract

## Background

Obesity is the epidemic of our time, with sharply and steadily rising rates [1,2]. The major adverse consequences of obesity including type 2 diabetes, atherosclerosis and essential hypertension, when added together, account for a large number of disease related deaths [3,4]. If the obesity-related cancer cases are added to this number, obesity-related mortality by far exceeds that of other common diseases [5]. The latter indicates the urgent need to develop novel efficient therapeutic modalities for this condition.

The common denominator in the pathogenesis of the co-morbidities of obesity is the presence of an active, low-grade inflammatory process [6]. Despite evidence linking obesity to alterations in inflammatory response, little is known about the specific effects of obesity on the immune system. Recently, there has been a greater appreciation of the role of epigenetics, meiotically and mitotically heritable changes in gene expression that are not coded in the DNA sequence itself, in the immune and inflammatory responses [7-9]. Therefore, we hypothesize that DNA methylation changes play a role in obesity induced immune dysfunction. The goal of this study was to characterize DNA methylation profile in peripheral blood leukocytes in obese versus lean subjects using a genome wide approach. Identification of methylation changes in specific genes will provide important targets for further study into the mechanisms of obesity's effect on the immune system and the potential to develop new therapies to treat multiple obesity comorbidities.

## Methods

### Subjects

The genome wide methylation analysis was conducted in seven obese and seven age-matched lean controls. These 14 subjects were identified from the participants (n = 534) in the Lifestyle, Adiposity, and Cardiovascular Health in Youth (LACHY) study using the following inclusion criteria: (1) African American (AA) ancestry; (2) male; (3) having leukocyte DNA available; (4) obese cases having a body mass index (BMI) ≥ 99^th ^percentile for age and sex and lean controls having BMI ≤ 10^th ^percentile for age and sex. The LACHY study consisted of roughly equal numbers of AA and European American (EA) adolescents aged 14 to 18 years of both sexes recruited from high schools in the Augusta, Georgia area [10].

The replication cohort included 46 obese (BMI ≥ 30 kg/m^2 ^or BMI ≥ 95^th ^percentile for age and sex if age ≤ 18) and 46 lean (BMI ≤ 22 kg/m^2 ^or BMI ≤ 40^th ^percentile for age and sex if age ≤ 18) AA males selected from three cohorts, the Blood Pressure (BP) stress study (n = 603) [11], the Georgia Cardiovascular twin study (n = 1,183) [12] and the Prevention of Hypertension in African American Teens (PHAT) study (n = 262) [13]. Both the BP Stress study and the twin study are on-going longitudinal studies which have followed the subjects more than 10 years. Both studies included roughly equal numbers of AAs and EAs or males and females. The BP stress study was established in 1989 with subjects aged to 7 to 16 years at baseline and the twin study was established in 1996 with subjects aged 7 to 25 years at baseline [11,12]. The PHAT study was a cross-sectional study and consisted of AA males and females aged 14 to 20 years [13]. Subjects in all the three studies were also recruited from Augusta, GA area. The obese and lean subjects were identified based on the following criteria: (1) having leukocyte DNA available; (2) AA males; (3) only one twin subject from a twin pair was selected if both twins met the criteria; (4) if multiple visits (with multiple leukocyte DNA) were available for a subject, this subject had to be obese or lean on all the visits to be included in the replication sample and the leukocyte DNA collected at the visit when the subject were the most obese or most lean was used.

For all four cohorts self identification by self-reports of each subject or by a parent if the subject was under 18 years of age was used to classify ethnicity according to previously described criteria [14]. Subjects in all the four studies were overtly healthy, free of any acute or chronic illness on the basis of parental reports and were not on anti-hypertensive, lipid lowering, anti-diabetic and anti-inflammatory medications [10-13]. The Institutional Review Board at the Medical College of Georgia approved the studies. Informed consent was obtained from all subjects and by parents if subjects were less than 18 years of age.

### Measurements

For all the four cohorts, height and weight were measured by standard methods using a wall-mounted stadiometer and a scale, respectively. BMI was calculated as weight/height^2^. Systolic BP (SBP) and diastolic BP (DBP) were measured with Dinamap monitors, using an appropriately sized BP cuff placed on the subject's right arm. BP measurements were taken at 11, 13, and 15 minutes, during a 15-minute supine relaxation period. The average of the last two readings was used to represent SBP and DBP values [10-13].

Fasting peripheral blood samples in the LACHY cohort and non-fasting peripheral blood samples in the other three cohorts were collected. The buffy coat and plasma samples were separated and stored at -80°C. DNA was extracted from the buffy coat. In the LACHY cohort, fasting glucose levels were measured using Ektachem DT II system (Johnson and Johnson Clinical Diagnostics, Rochester, NY, USA) and fasting insulin was assayed in duplicate by specific radioimmunoassay (Linco Research, Inc., St Charles, MO, USA) [10]. QUICKI (quantitative insulin-sensitivity check index) was calculated to index insulin sensitivity using the following formula: 1/[log(fasting insulin, μU/ml) + log(fasting glucose, mg/dl)]. Out of the screening sample which included the seven obese cases and seven lean controls selected from the LACHY cohort, fasting glucose and insulin levels were not available for one case and one control.

### Genome wide methylation chip

The HumanMethylation27 BeadChip from Illumina (Illumina, San Diego, CA, USA) was used. This chip can quantitatively measure 27,000 CpG sites, covering more than 14,000 well-annotated genes at single-CpG resolution. Each chip can accommodate 12 samples. After bisulfite treatment, 200 ng of the converted DNA was whole genome amplified (WGA) and enzymatically fragmented. The bisulfite-converted WGA-DNA samples were purified and applied to the BeadChips. Image processing and intensity data extraction were performed according to Illumina's instruction http://www.illumina.com/products/infinium_humanmethylation27_beadchip_kits.ilmn. Each methylation data point is represented by fluorescent signals from the methylated and unmethylated alleles. DNA methylation beta values are continuous variables between 0 (completely unmethylated) and 1 (completely methylated), representing the ratio of the intensity of the methylated bead type to the combined locus intensity. Initial array processing and quality control were performed with BeadStudio software. The microarray data discussed in this paper have been deposited in NCBI's Gene Expression Omnibus and are accessible through GEO Series accession number GSE25301 http://www.ncbi.nlm.nih.gov/geo/query/acc.cgi?acc=GSE25301.

### Pyrosequencing

The methylation levels of the selected six CpG sites from the six genes in the replication cohort were determined by pyrosequencing technology, a rapid and robust method for quantitative methylation analysis. After bisulfite treatment, 10 ng of the converted DNA was used in a PCR reaction to amplify the target region. One of the PCR primers was biotin labeled. Single-stranded biotinylated PCR products were prepared for sequencing by use of the Pyrosequencing Vacuum Prep Tool according to the manufacturer's instructions. The PCR products (each 10 μl) were sequenced by Pyrosequencing PSQ96 HS System (Pyrosequencing-Qiagen) following the manufacturer's instructions. The methylation status of each locus was analyzed individually as a T/C SNP using QCpG software (Biotage, Kungsgatan, Sweden). PCR primers and sequencing primers for these six genes are listed in Additional file 1. All the samples were assayed in the same run and in a random sequence.

### Statistical analysis

For the genome wide methylation analysis, the Limma package [15] was used to analyze each CpG site for differential methylation between obese and lean subjects. CpG sites on the × and Y chromosomes were excluded from the analysis. Each CpG site was assigned a raw *P*-value based on a moderated t statistic. To correct for multiple testing, the set of raw *P*-values were converted to false discovery rates (FDR) according to Benjamini and Hochberg [16]. For the six CpG sites in the replication cohort, a Student's *t*-test was used to investigate whether their methylation levels differ between the obese and the lean group. Linear regression was further used to adjust the potential effect of age on methylation levels. We combined the genome wide and the replication steps for these six CpG sites using the weighted z score-based meta-analysis approach implemented in the package METAL [17]. Prior to analysis, methylation levels of the CpG sites in the replication cohort were log-transformed or square root-transformed to obtain a better approximation of the normal distribution. Preliminary analyses, t-tests and regression analyses were done using STATA 8 (StataCorp, College Station, TX, USA).

Gene Ontology analysis was conducted with the FatiGO tool [18]. FatiGO takes two lists of genes and converts them into two lists of GO terms. Then a Fisher's exact test for 2 × 2 contingency tables is used to check for significant over-representation of GO terms in one of the sets with respect to the other one. Multiple testing correction (indexed by adjusted *P-*values) to account for the multiple hypothesis tested (one for each GO term) is applied to reduce false positives. Since at least two CpG sites were included for the majority of genes in this genome wide chip, we selected the CpG sites with the lowest p value to represent this gene.

## Results

**Figure 1:**
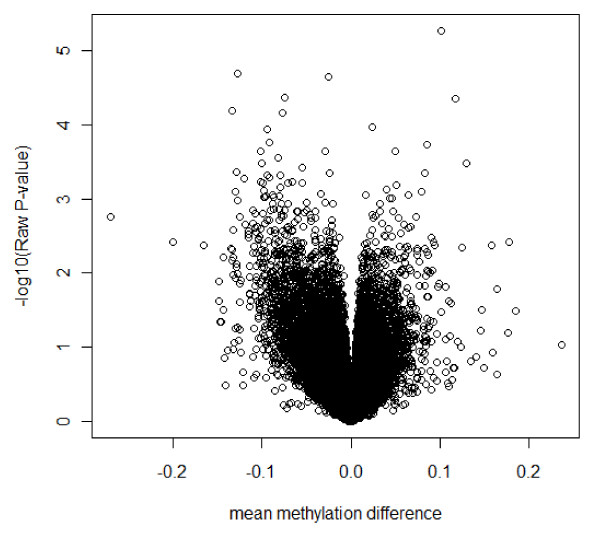
**Volcano plot showing raw *P-*values versus mean methylation difference between obese cases and lean controls**.

Table 1 displays the general characteristics of the screening sample. At this age range, obese subjects already showed significantly higher insulin resistance. Figure 1 is a volcano plot showing the raw *P*-values for all CpG sites versus mean methylation difference between the obese group and the lean group. We did not observe any CpG sites survive multiple testing correction with the most significant CpG site with a FDR of 0.14 and a raw *P-*value of 5 × 10^-6^. Table 2 lists the top 10 most significant CpG sites and one additional CpG site which showed the largest methylation difference between cases and controls (-27.1% for *CREB3L3*). Since at least two CpG sites were included for the majority of the genes in this genome wide methylation chip, the *P*-value for the second CpG site in this gene was also listed in Table 2. Out of the 11 CpG sites listed in Table 2 (under the column "more significant CpG site"), we selected six CpG sites for validation in the replication cohort using the following inclusion criteria: (1) the most significant differentially methylated CpG site, which is the CpG site locating at -156 of the *UBASH3A *(ubiquitin-associated and SH3 domain-containing protein A) gene; or (2) the CpG sites in those genes of which the second CpG site also showed a raw *P *< 0.05. These include the CpG sites from the *TRIM3 *(Tripartite motif-containing 3), *CTSZ *(Cathepsin Z preproprotein), *HIPK3 *(Homeodomain interacting protein kinase 3), *CDH5 *(Cadherin 5 type 2 preproprotein) and *CREB3L3 *(cAMP responsive element binding protein 3-like 3) genes. The validation was conducted on the more significant CpG site (with Illumina ID 13517, 17029, 23241, 25599, 8829 and 23739) in each gene by pyrosequencing. The general characteristics of the replication cohort are displayed in Table 3. Since the DNA from one obese subject failed on four pyrosequencing assays, the data from this subject was excluded from the analysis. Table 4 lists the results in the replication cohort. The findings from the genome wide methylation chip were validated for the CpG sites in the UBASH3A, TRIM3, HIPK3 and CDH5 genes. The methylation status of the CpG site in the UBASH3A gene was 3.3% higher (*P *= 0.0019) and the methylation status of the CpG site in the TRIM3, HIPK3 and CDH5 genes was 1.21% (*P *= 0.0004), 7.31% (*P *= 0.0135) and 3.09% (*P *= 0.045) lower in the obese versus the lean group. These significant findings persisted after adjustment of age for the association between obesity and methylation status of the CpG site in the UBASH3A gene (*P *= 0.008) and TRIM3 gene (*P *= 0.001), but changed to borderline significance for HIPK3 gene (*P *= 0.05), and disappeared for the CpG site in the CDH5 gene (*P *= 0.095). The results of the meta-analysis for these six CpG sites on the data from the genome wide step and the replication step are shown in Additional file 2

**Table 1:** General characteristics of the subjects for genome wide methylation analysis

	Cases	Controls
N	7	7
Age, years	15.8 ± 1.0 (14.5 to 17.2)	15.9 ± 1.4 (15.1 to 18.1)
*BMI, kg/m^2^	39.0 ± 1.7 (37.2 to 41.2)	17.0 ± 0.7 (16.4 to 17.8)
*BMI percentile	0.996 ± 0.001 (0.995 to 0.997)	0.048 ± 0.022 (0.026 to 0.091)
SBP, mmHg	125.8 ± 13.9 (115.3 to 152.7)	112.7 ± 8.9 (100.7 to 124.7)
^†‡^Fasting insulin, uU/ml	35.9 ± 13.6 (19.8 to 58.9)	13.7 ± 3.0 (9.4 to 17.0)
^‡^Fasting glucose, mg/dl	103.5 ± 14.8 (84 to 126)	88.8 ± 7.6 (80 to 100)
*^‡^QUICKI	0.28 ± 0.01 (0.27 to 0.30)	0.33 ± 0.01 (0.31 to 0.35)

Means ± SD (Range)

*: *P *< 0.001; †: *P *< 0.01

‡: Available for six cases and six controls

**Table 2:** Top 10 differentially methylated CpG sites and the CpG site showing the largest difference.

Gene	More significant CpG site						Second CpG site				
		
	ID	Dis. to TSS	Average Case/Con. (%)	Difference (%)	*P*	FDR	ID	Dis. to TSS	Average Case/Con. (%)	Difference (%)	*P*
**UBASH3A**	13517	-156	45.6/35.5	10.1	5.00 × 10^-6^	0.14	146	52	45.6/37.7	7.9	0.145
CTNND1	6160	-431	57.4/70.2	-12.8	2.00 × 10^-5^	0.20	18016	415	19.7/21.5	-1.8	0.353
**TRIM3**	17029	-348	6.74/9.28	-2.54	2.40 × 10^-5^	0.20	19207	-331	12.5/16.2	-3.7	0.035
CTSZ*	23241	-292	13.6/21.0	-7.40	4.40 × 10^-5^	0.24	1644	-592	17.4/25.6	-8.2	0.001
TRAF5	10097	-319	69.7/58.0	11.7	4.50 × 10^-5^	0.24	25786	77	1.90/1.79	0.1	0.738
**HIPK3**	25599	390	51.5/64.9	-13.4	6.50 × 10^-5^	0.26	5514	314	26.6/35.0	-8.4	0.006
DPCR1	20918	-46	62.5/70.2	-7.70	6.90 × 10^-5^	0.26	4703	-906	72.4/73.0	-0.6	0.709
HIF3A	2879	153	5.35/2.97	2.38	1.08 × 10^-4^	0.34	7009	-1445	57.1/63.0	-5.9	0.091
NOTCH4	5969	-51	59.1/68.5	-9.40	1.15 × 10^-4^	0.34	14700	4	19.3/23.6	-4.3	0.078
**CDH5**	8829	-243	27.2/36.5	-9.20	1.75 × 10^-4^	0.44	22286	36	72.5/81.3	-8.8	0.012
**CREB3L3**	23739	42	9.63/36.7	-27.1	1.78 × 10^-3^	0.59	13445	-26	5.74/25.8	-20.1	0.004

Genes taken forward to the replication stage are shown in **bold**.

ID: ID from Illumina HumanMethylation27 BeadChip; Dis. to TSS: Distance to the Transcription Start Site; FDR: False Discover Rate

* For CTSZ, 7 CpG sites were included in the genome-wide methylation platform and the two most significant sites are listed in the table.

**Table 3:** General characteristics of the subjects of the replication cohort

	Cases	Controls
N	45	46
*Age, years	20.3 ± 5.0 (14.1 to 29.5)	17.6 ± 3.1 (12.7 to 27.9)
^†^BMI, kg/m^2^	37.4 ± 6.1 (30.5 to 59.9)	18.9 ± 1.2 (16.4 to 22.0)
^†^SBP, mmHg	125.8 ± 14.2 (97 to 175)	113.5 ± 8.5 (100 to 134)

Means ± SD (Range)

*: *P *< 0.01; †: *P *< 0.001

**Table 4:** Results from the replication cohort

Gene	ID	Dis. to TSS	Average Case/Con. (%)	Difference (%)	*P*	Age adjusted *P*
UBASH3A	**13517**	**-156**	**45.1/41.8**	**3.30**	**0.0019**	**0.008**
TRIM3	---	-357	8.58/8.98	-0.40	0.3128	0.059
	**17029**	**-348**	**8.76/9.97**	**-1.21**	**0.0004**	**0.001**
	19207	-331	6.91/7.93	-1.02	0.0050	0.022
CTSZ	**23241**	**-292**	**16.5/17.7**	**-1.20**	**0.0953**	**0.271**
	---	-279	27.8/29.1	-1.30	0.8425	0.798
HIPK3	---	375	48.0/55.3	-7.30	0.0184	0.061
	**25599**	**390**	**49.8/57.1**	**-7.30**	**0.0135**	**0.050**
	---	422	39.8/45.8	-6.00	0.0164	0.070
	---	438	42.5/49.0	-6.50	0.0173	0.061
CDH5	**8829**	**-243**	**30.3/33.4**	**-3.10**	**0.0450**	**0.092**
CREB3L3	---	30	3.23/2.85	0.38	0.7188	0.828
	**23739**	**42**	**18.7/21.6**	**-2.90**	**0.3251**	**0.288**
	---	48	18.5/22.3	-3.80	0.1549	0.174

ID, ID from Illumina HumanMethylation27 BeadChip

Dis. to TSS, Distance to the Transcription Start Site.

The targeted CpG sites are highlighted in bold.

Although the pyrosequencing assay was designed to target one specific CpG site for each gene, some of the assays covered several surrounding CpG sites. The results of these CpG sites are also listed in Table 4. The correlation within samples among the multiple CpG sites measured within a gene is listed in Additional file 3.

Gene Ontology analysis was performed to test whether some common functional trends in molecular functions and biological processes were associated with the genes exhibiting differences between obese cases and lean controls in the genome wide chip. We included those genes with a raw *P *≤ 0.01 to the first list (n = 298) and included all the other genes in the second list. As expected from a pilot study in 14 subjects, we did not observe any GO categories survive multiple testing. Table 5 lists the GO categories with raw *P*-value less than 0.05. Interestingly, we observed enriched functional processes that are potentially relevant for inflammatory response with immune response (GO:0006955), cell activation (GO:0001775), cytokine production (GO:0001816), response to biotic stimulus (GO:0009607) and antigen binding (GO:0003823) among the top GO categories. The results support that obesity might lead to methylation changes in genes involved in inflammatory pathways.

**Table 5:** Gene-ontology Analysis

Biological process	GO. ID	Term	*P- *value	Adjust *P*
Level 3	GO:0006955	Immune response	0.017	0.690
Level 3	GO:0007588	Excretion	0.028	0.690
Level 3	GO:0001775	Cell activation	0.042	0.690
Level 3	GO:0001816	Cytokine production	0.044	0.690
Level 3	GO:0009607	Response to biotic stimulus	0.047	0.690

Molecular function	GO. ID	Term	*P *value	Adjust *P*

Level 3	GO:0019842	Vitamin binding	0.009	0.522
Level 3	GO:0003823	Antigen binding	0.010	0.522
Level 3	GO:0004872	Receptor activity	0.032	0.756
Level 3	GO:0008289	Lipid binding	0.035	0.756

## Discussion

In this study we aimed to identify obesity related methylation changes in peripheral blood leukocytes using a genome wide approach in youth and young adults who are free of obesity comorbidities. The primary findings of this study are increased methylation levels at one CpG site in the *UBASH3A *gene and decreased methylation level at one CpG site in *TRIM3 *gene in obese subjects compared with lean controls.

The protein encoded by the *UBASH3A *gene is the "ubiquitin-associated and SH3 domain-containing protein A", which was previously also known as T-cell ubiquitin ligand (TULA) and suppressor of T-cell signaling 2 (Sts-2). It is expressed predominantly in T-cells, where it has a suppressing effect on T-cell signaling and activation. It acts in part by inhibiting c-CBL-mediated downregulation of protein tyrosine kinases (PTKs) that are activated upon T-cell receptor stimulation [19]. UBASH3A is also capable of promoting T-cell apoptosis [20]. *UBASH3A *and *UBASH3B *(another protein in this family) gene double knock-out mice were hyperresponsive to T-cell receptor stimulation with increased cytokine secretion, although mice lacking *UBASH3A *were normal in all respects including T-cell function [21]. However, whether the expression of the *UBASH3A *gene is regulated by DNA methylation is unknown. No CpG island exists in this gene and the CpG site showing higher methylation levels in obese subjects from this study locates at the promoter region with a distance of 156 bp to the transcription start site. There is a possibility that methylation of this CpG site or other CpG sites with methylation levels correlated with this CpG site inhibits the interactions between DNA sequence and nuclear proteins, resulting in decreased gene expression. In-silico analysis of the region of this CpG site using TFSEARCH software [22] did not find this CpG site located at any known transcription factor binding sites. However, methylation of this CpG site may suppress gene transcription by recruiting methylcytosine-binding proteins that in turn associate with large protein complexes containing corepressors and histone deacetylases. The binding of these complexes to DNA may lead to a change in the chromatin structure from an active to an inactive form [23]. This speculation needs to be confirmed.

*TRIM3 *is one member of the TRIM protein family. These proteins share a conserved tripartite architecture and have a variety of cellular functions including cell proliferation, differentiation, oncogenesis, apoptosis, immune signaling and have been implicated in autoimmune diseases [24]. In peripheral blood leukocytes, *TRIM3 *is mainly expressed in macrophages and can be up-regulated by viral infection [25,26]. Similar to *UBASH3A*, the role of methylation in *TRIM3 *gene expression has not been reported yet. The *TRIM3 *gene has 1 CpG island located in the promoter and exon 1 (nucleotides -446 to 576), one CpG island spanning intron 2 and exon 3 (nucleotides 7759 to 8334), and one CpG island in exon 13 and the downstream region (nucleotides 24883 to 25610). The CpG site showing lower methylation levels in obese subjects in both the scanning cohort and the replication cohort locates in the promoter CpG island (348 bp upstream to the transcription start site). The methylation status of the other CpG site which showed significant differential methylation levels between obese cases and lean controls in the genome wide methylation analysis also locates in this CpG island (331 bp upstream to the transcription start site). Because the pyrosequencing assay also measured the methylation levels of the -331 CpG site (Illumina ID: 19207), we were able to show that also for this site obese subjects had lower methylation levels in comparisons with the lean subjects (age adjusted *P *= 0.022 as shown in Table 4). Similar to the CpG site in the *UBASH3A *gene, these two CpG sites are not located at any known transcription factor binding sites. Future studies will be needed to test whether the density of the methylation or the methylation of specific CpG sites in this CpG island has effect on *TRIM3 *gene expression.

The observed DNA methylation differences between obese cases and controls were relatively small. They were 10.1% and 2.54% in the genome wide step and 3.3% and 1.2% in the replication step for the UBASH3A gene and the TRIM3 gene, respectively. This modest level of differences raises an important question: what is the biological significance of changes in methylation on this degree? In this study, we used the DNA from leukocytes, which represent different cell populations with distinct epigenetic profiles. It is plausible that only the change in the epigenetic profile of one specific cell type is related to obesity. In this regard, the actual epigenetic differences might be more substantial than reported here but only present in this specific blood leukocyte cell type. Future studies on epigenetic profiling of various types of cell populations in the leukocytes are warranted to gain a greater understanding of the epigenetic dysregulation in obesity. Furthermore, this kind of study will be able to identify the cells specifically reflecting obesity-associated methylation changes, which is of great interest in itself. Transcriptional profiling studies will be very valuable in understanding whether the DNA methylation status differences are associated with differences in gene expression. Unfortunately, cellular RNA is not available for the samples used in the current study, which were selected from several existing cohorts.

There are several strengths of this study. First, we selected obese cases and controls with extreme phenotypes, which maximizes the power to make discoveries. Second, we focused on youth and young adults with the distinct advantage that the results are not confounded by obesity comorbidities or use of medication, both of which are very common in adult subjects with obesity. Third, a hypothesis-free genome wide approach was used. This approach supersedes the limitations imposed by candidate gene methylation studies and allows searching the whole genome in an unbiased manner.

Interpretation of these data is also limited by several constraints. First, in this study we hypothesize that obesity will lead to methylation changes in the DNA of leukocytes, which further lead to obesity related co-morbidities. However, our study design cannot determine whether the identified methylation changes are the cause or the consequence of obesity. Future studies on subjects who changed their body size status will be needed to clarify the causal directions. On the other hand, epigenetic regulation is tissue specific. In this regard, the target tissue to identify epigenetic variations responsible for obesity should be the hypothalamus of the brain rather than peripheral leukocytes. Second, in the current study, the Infinium HumanMethylation27 Beadchip was selected because of its quantitative measure at each CpG site. However, the limited coverage of this genome wide chip will restrict the findings to certain CpG sites within certain genes. Future studies should use chips with more complete coverage of the genome such as the recently released 450K Infinium Methylation BeadChip from Illumina. Third, the current study is a small pilot study with the genome wide step conducted only in seven obese and seven lean subjects. Future studies with much larger sample size are warranted to discover a more complete profile of obesity related methylation changes. Fourth, although all the obese cases in the genome-wide step were free of clinical diseases such as CVD and diabetes, some of them already showed evidence of insulin resistance. It is possible that insulin resistance may be the factor triggering methylation changes and not the obesity *per se*. Recent literature supports the postulate that glucose restriction alters gene expression through epigenetic mechanisms [27]. We conducted further analysis within the obese cases and did not observe that any of the CpGs showed methylation difference between cases with impaired fasting glucose (n = 3) versus those with normal glucose levels (n = 3), with the most significant CpG site having an adjusted *P-*value of 0.85. However, the limited sample size prevents us from drawing any conclusion based on this analysis. Unfortunately, fasting glucose was not measured in the replication cohort. Future studies with large sample sizes and more detailed phenotypes will be needed to clarify this question.

## Conclusion

In this study, we identified several reproducible changes in DNA methylation of peripheral blood leukocytes between obese cases and lean controls. This study provides evidence that obesity is associated with methylation changes in blood leukocyte DNA. Further studies are warranted to determine the causal direction of this relationship as well as whether such methylation changes can lead to immune dysfunction. Such studies will have the ability to identify new insight into disease etiology and provide new targets for prevention of obesity related diseases such as cardiovascular diseases and type 2 diabetes.

## Abbreviations

AA: African American; BMI: Body Mass Index; BP: Blood Pressure; CDH5: Cadherin 5 type 2 preproprotein; CREB3L3: cAMP responsive element binding protein 3-like 3; CTSZ: Cathepsin Z preproprotein; DBP: Diastolic Blood Pressure; EA: European American; FDR: False Discovery Rate; HIPK3: Homeodomain interacting protein kinase 3; LACHY: Lifestyle, Adiposity, and Cardiovascular Health in Youth; PHAT: Prevention of Hypertension in African American Teens; QUICKI: Quantitative Insulin-Sensitivity Check Index; SBP: Systolic Blood Pressure; TRIM3: Tripartite motif-containing 3; UBASH3A: Ubiquitin-associated and SH3 domain-containing protein A; WGA: Whole Genome Amplified.

## Competing interests

The authors declare that they have no competing interests.

## Authors' contributions

XW had full access to all of the data in the study and takes responsibility for the integrity of the data and the accuracy of the data analysis. XW and HS contributed to the study concept and design. XW, SS, HS, HS and DM analyzed and interpreted the data. HZ, YD, BG, FT and GH acquired the data. XW drafted the manuscript. XW, HS, GH and BLM critically revised the manuscript for important intellectual content. XW and SS did the statistical analysis.

## Pre-publication history

The pre-publication history for this paper can be accessed here:

http://www.biomedcentral.com/1741-7015/8/87/prepub

## Supplementary Material

Additional file 1 **Primer sequences and PCR conditions for bisulfit-pyrosequencing analysis**. Click here for file

Additional file 2 **Meta-analysis for the six CpG sites**. Click here for file

Additional file 3 **Correlations among the multiple CpG sites within one gene**. Click here for file
