# Supplementary material for: Obesity related methylation changes in DNA of peripheral blood leukocytes
Source: BMC Med. 2010 Dec 21;8:87. doi: 10.1186/1741-7015-8-87 (PMC3016263; doi:10.1186/1741-7015-8-87)
Supplement: Additional file 2 — Meta-analysis for the six CpG sites. [file 1741-7015-8-87-S2.DOC]

Additional file 2. Meta-analysis for the 6 CpG sites

| Gene | ID | P value |
| --- | --- | --- |
| UBASH3A | 13517 | 5.10×10^-5^ |
| TRIM3 | 17029 | 5.68×10^-6^ |
| CTSZ | 23241 | 1.51×10^-2^ |
| HIPK3 | 25599 | 1.35×10^-3^ |
| CDH5 | 8829 | 4.18×10^-3^ |
| CREB3L3 | 23739 | 3.89×10^-2^ |

ID: ID from Illumina HumanMethylation27 BeadChip
