# Supplementary material for: Obesity related methylation changes in DNA of peripheral blood leukocytes
Source: BMC Med. 2010 Dec 21;8:87. doi: 10.1186/1741-7015-8-87 (PMC3016263; doi:10.1186/1741-7015-8-87)
Supplement: Additional file 3 — Correlations among the multiple CpG sites within one gene. [file 1741-7015-8-87-S3.DOC]

Additional file 3. Correlations among the multiple CpG sites within one gene

| Additional file 3a. TRIM3 | | | |
| --- | --- | --- | --- |
| Distance to TSS | Distance to TSS | | |
|  | -357 | -348 | -331 |
| -357 | 1 |  |  |
| -348 | 0.6095^*^ | 1 |  |
| -331 | 0.5025^*^ | 0.5538^*^ | 1 |

| Additional file 3b. HIPK3 | | | | |
| --- | --- | --- | --- | --- |
| Distance to TSS | Distance to TSS | | | |
|  | 375 | 390 | 422 | 438 |
| 375 | 1 |  |  |  |
| 390 | 0.9868^*^ | 1 |  |  |
| 422 | 0.9827^*^ | 0.9848^*^ | 1 |  |
| 438 | 0.9794^*^ | 0.9728^*^ | 0.9719^*^ | 1 |

| Additional file 3c. CREB3L3 | | | |
| --- | --- | --- | --- |
| Distance to TSS | Distance to TSS | | |
|  | 30 | 42 | 48 |
| 30 | 1 |  |  |
| 42 | 0.0678 | 1 |  |
| 48 | 0.0219 | 0.9633^*^ | 1 |

*P<0.001
