# Supplementary material for: Obesity related methylation changes in DNA of peripheral blood leukocytes
Source: BMC Med. 2010 Dec 21;8:87. doi: 10.1186/1741-7015-8-87 (PMC3016263; doi:10.1186/1741-7015-8-87)
Supplement: Supplementary file 3 [file 1741-7015-8-87-S1.DOC]

Additional file 1. Primer sequences and PCR conditions for bisulfit-pyrosequencing analysis

	PCR			Pyrosequencing	
Gene	Forward primer	Reverse primer	Size (bp)	Sequence primer	Sequence region
CTSZ	GAATGTAGGGTTAAGGGGGTTTATG	CTCCCTAACTCCRAAACACCTA	182	GAATGTAGGGTTAAGGGGGTTTATG	AGTTTGYGGTTTTTTATTTYGAAGTTAGGAATTT
UBASH3A	GTTAGTAGGATTTAGGAGGGAAT	ACATAAAATATCAATTTTCAAAATTCT	231	GGTGGAAATAGTTAGCATTGG	TGGAYGTTGTATTTAGTATTTTGTAAATAG
TRIM3	AGTATATTTTATAGGAGTATTTTAAGGAA	ACCCTACCTCCTTAATACTACTAAA	254	TTATAGGAGTATTTTAAGCAAGT	TYGGAAAGGGYGTTTTTAGGAGGTAGGYGGTTTAGATAGGTTTTTAGAGTT
CDH5	AGGAGGGTTAAATGTGATGTTGTA	CCCACTTCCCCCTAAAATAATT	255	GGTTTAGAGTTTTAGAGTTG	AGGTYGGGAGTAGGGGTTTAAGT
CREB3L3	GGTTGGGAGTTGTTTGTGAT	AAAATCCTTCCAACTCTAACAACTTA	270	GGTGATAGAGTTATAGAGGG	TTGTGAGTTTGTTYGGTTTTAGGTAAYGTTGGYGGTGGGTGGGTTTTTAGTTTGGAGT
HIPK3	TTGAAAAATATTGTAGGTGTTATAAAGGTTA	TCACTAAATTTCCCATATTAATTTACAA	235	TGTATTTTTTAGAAGGTTTT	TAGYGATGTGGATTGAAGYGTAAGAGTGAGGAGTTGGATAATTATAGTAGYGTAATGTAGATTGTYGATGAATTGTTTATATTTTTTGTAA
